# C-Terminal Amino Acids 471-507 of Avian Hepatitis E Virus Capsid Protein Are Crucial for Binding to Avian and Human Cells

**DOI:** 10.1371/journal.pone.0153723

**Published:** 2016-04-13

**Authors:** Xinquan Zhang, Ivana Bilic, Ana Marek, Martin Glösmann, Michael Hess

**Affiliations:** 1 Clinic for Poultry and Fish Medicine, Department for Farm Animals and Veterinary Public Health, University of Veterinary Medicine, Vienna, Austria; 2 VetCore Facility for Research, University of Veterinary Medicine, Vienna, Austria; Virginia Polytechnic Institute and State University, UNITED STATES

## Abstract

The infection of chickens with avian Hepatitis E virus (avian HEV) can be asymptomatic or induces clinical signs characterized by increased mortality and decreased egg production in adult birds. Due to the lack of an efficient cell culture system for avian HEV, the interaction between virus and host cells is still barely understood. In this study, four truncated avian HEV capsid proteins (ORF2-1 – ORF2-4) with an identical 338aa deletion at the N-terminus and gradual deletions from 0, 42, 99 and 136aa at the C-terminus, respectively, were expressed and used to map the possible binding site within avian HEV capsid protein. Results from the binding assay showed that three truncated capsid proteins attached to avian LMH cells, but did not penetrate into cells. However, the shortest construct, ORF2-4, lost the capability of binding to cells suggesting that the presence of amino acids 471 to 507 of the capsid protein is crucial for the attachment. The construct ORF2-3 (aa339-507) was used to study the potential binding of avian HEV capsid protein to human and other avian species. It could be demonstrated that ORF2-3 was capable of binding to QT-35 cells from Japanese quail and human HepG2 cells but failed to bind to P815 cells. Additionally, chicken serum raised against ORF2-3 successfully blocked the binding to LMH cells. Treatment with heparin sodium salt or sodium chlorate significantly reduced binding of ORF2-3 to LMH cells. However, heparinase II treatment of LMH cells had no effect on binding of the ORF2-3 construct, suggesting a possible distinct attachment mechanism of avian as compared to human HEV. For the first time, interactions between avian HEV capsid protein and host cells were investigated demonstrating that aa471 to 507 of the capsid protein are needed to facilitate interaction with different kind of cells from different species.

## Introduction

Beside asymptomatic infections, avian hepatitis E virus (avian HEV) has been identified as etiological agent of two syndromes: big liver and spleen disease and hepatitis-splenomegaly syndrome [1]. Big liver and spleen disease was first recognized in Australia in the 1980s as an economically important disease of broiler breeders. Almost in parallel, hepatitis–splenomegaly syndrome was described in the United States as a disease that causes slightly increased mortality and decreased egg production in broiler breeders and laying hens [2,3]. Transmission of the virus occurs through the fecal-oral route but vertical transmission has been suggested as well [4]. The presence of avian HEV has been widely detected around the world like in China [5], Australia [6], Korea [7], United States [8] and Europe [6,9,10].

The avian HEV species with its 4 genotypes have been proposed to form an individual genus, designated *Avihepevirus*, within the family of *Hepeviridae* [11]. Other Hepatitis E viruses found in mammals and rodents belong to the same family, but are assigned to different genera [11]. Avian HEV is a non-enveloped positive single stranded RNA virus with a genome size of approximate 6.6Kb excluding the 3´ poly(A) tail [12]. The genome organization of all HEV species is similar, with slight differences in the total length and position of the open reading frames (ORF) 1–3. Similar to mammalian HEV, the genome is mainly organized in 3 ORFs, with non-coding regions of 24 and about 130 nt at the 5’- and 3’-end, excluding a poly(A) tail, respectively. ORF1 is located at 5’-end and encodes a polyprotein containing methyltransferase, papain-like cysteine protease, helicase and RNA-dependent RNA polymerase. It overlaps neither with ORF2 nor ORF3 and includes a hypervariable region of around 50 amino acids in length [13,14]. Following the stop codon of ORF1, there is a short non-coding region, which may play an important role in viral replication [15]. ORF3, which partially overlaps with ORF2, encodes a small phosphoprotein. ORF1 and ORF3 encode non-structural proteins which play a very important role for the replication of the virus [16]. ORF2 is located at the 3’-end of the genome and encodes the capsid protein with a length of 606 amino acids.

In previous reports, several infectious cDNA clones of avian HEV have already been constructed and proven to be a useful tool for in *vivo* studies [17–19]. In addition to that, the development and application of a detection system for negative-strand viral RNA provided valuable information about virus replication sites in *vivo* [20]. Even though these research efforts have already substantially improved our understanding on avian hepatitis E virus and host interaction, the molecular mechanism of virus attachment and entry is still not known.

In general, attachment of the virus to the host cell is considered a crucial step in viral infection, and differences in the mechanism of virus attachment among viruses from the same family have been widely observed [21,22]. Currently, there are no robust cell culture systems available for both mammalian and avian HEV, which complicates investigations addressing the interaction between virus and host. Consequently, in earlier studies a series of recombinant capsid proteins of human HEV were used to elucidate virus—hosts interactions. These studies showed that heparan sulfate (HS) proteogylcans were recognized as molecules required for the attachment of human HEV to host cells [23–25] and that the capsid C-terminal region includes binding sites for host cell receptors [26]. However, nothing is known about the binding of avian HEV to host cells. Therefore the aim of this study was to investigate virus-host interaction of avian HEV utilizing recombinant capsid contruct(s) as a replacement for mature virions. A series of truncated recombinant avian HEV capsid proteins were tested for binding to LMH cells and to map the potential binding region within avian HEV capsid protein. Binding to cells from other species was assessed in binding assay using cells derived from humans and Japanese quail. Finally, to elucidate whether HS proteoglycans on the host-cell surface are required for binding of the avian HEV, assays with heparin sodium salt, sodium chlorate and heparinase II were performed.

## Materials and Methods

### Cell cultures

The following cell lines were used in this study: LMH (ATCC, CRL-2117), QT-35 [27], HepG2 and mouse mastocytoma P815 [28]. LMH cells were derived from chicken hepatocellular carcinoma epithelial cells and have been reported to support avian HEV replication [19]. The QT-35 cells were derived from chemically-induced fibrosarcoma of Japanese quail. Human hepatocellular carcinoma derived cells HepG2 supports propagation and passages of mammalian HEV [29]. HepG2 cells and P815 cells were kind gifts from Hans Tillmann Rümenapf and Armin Saalmüller, respectively (University of Veterinary Medicine, Vienna).

LMH cells and P815 were grown in RPMI Medium 1640 supplemented with 10% fetal bovine serum (FBS). QT-35 and HepG2 cells were cultured in Dulbecco's Modified Eagle Medium containing 10% FBS with or without 10% Tryptose Phosphate Broth, respectively. All media and supplements were purchased from Life Technologies (Carlsbad, CA, USA). All cells were grown at 37°C in an atmosphere supplied with 5% CO_2_.

### Expression and preparation of truncated recombinant capsid proteins

Viral RNA was extracted from liver homogenate of a chicken infected with a genotype 1 isolate of avian HEV sample number 05–2294 as described previously [12]. The ORF2-1 DNA fragment was amplified by RT-PCR using OneStep RT-PCR kit (Qiagen, Vienna, Austria) and primers ORF2-1 forward and ORF2-1 reverse (S1 Table). The other truncated fragments, ORF2-2, ORF2-3 and ORF2-4, were generated by conventional PCR using previously amplified ORF2-1 as template, ORF2-1 forward primer with corresponding reverse primers ORF2-2, ORF2-3 and ORF2-4, respectively (S1 Table).

Expression vector constructs were made with pRSET B (Invitrogen, Life Technologies, Carlsbad, CA, USA) and previously generated DNA fragments, by cloning them in frame with 6xHistidine- and Xpress-tags. All expression constructs were confirmed by sequencing.

The expression plasmids were transformed into *Escherichia coli* strain BL21 (DE3) (Invitrogen, Life Technologies, Carlsbad, CA, USA) and expression was induced by incubation with 0.5mM isopropyl β-D-1-thiogalactopyranoside (IPTG) for 3h. All expressed recombinant proteins formed inclusion bodies. Proteins in inclusion bodies were purified and refolded using the Pierce^™^ protein refolding kit (Pierce, Thermo Fisher Scientific, Life Technology, Carlsbad, CA, USA) according to the manufacturer´s instructions. Briefly, bacteria were collected by centrifugation and lysed in the presence of 500μg/ml lysozyme (Fluka, Buchs, Switzerland), then insoluble inclusion bodies were pelleted by centrifugation. Purified inclusion bodies were dissolved in 6M guanidine hydrochloride (Sigma-Aldrich, Steinheim, Germany) for denaturation, denatured recombinant proteins were refolded by rapid dilution in base refolding buffer (880mM L-arginine, 55mM Tris, 21mM NaCl, 0.88mM KCl; pH 8.2) with 10mM final concentration of EDTA, reduced glutathione and oxidized glutathione. Refolded proteins were dialyzed against PBS and stored at -80°C.

### SDS-PAGE and Western blotting

Truncated proteins were analyzed by sodium dodecyl sulfate-polyacrylamide gel electrophoresis (SDS-PAGE) and Western blotting. Briefly, each purified protein was separated by SDS-PAGE under reducing or non-reducing conditions, and then stained with Coomassie blue or transferred to polyvinylidene fluoride membrane (PVDF) (GE Healthcare, Little Chalfont, Bukinghamshire, UK). For immuno-detection, the membrane was saturated with 5% skimmed milk for 2h at room temperature (RT) and incubated with mouse monoclonal anti-His (Sigma-Aldrich, Steinheim, Germany) in 1:12500 dilution for 1h at RT. After washing, the membrane was incubated with horse radish peroxidase (HRP)-conjugated goat anti-mouse IgG in 1:12500 dilution for 1h at RT and proteins were detected with SuperSignal^™^ West Pico Chemiluminescent Substrate (Pierce, Thermo Fisher Scientific, Life Technology, Carlsbad, CA, USA).

### Binding assay

A binding assay was applied to investigate the binding of all truncated recombinant capsid proteins or PBS (negative control) to LMH cells. ORF2-3 and ORF2-4 constructs were selected for the binding assay using QT-35, HepG2 and P815 cells. Briefly, cells were grown to proper confluence and washed gently with PBS. Afterwards, cells were incubated with 500nM truncated recombinant capsid protein for 1h at 37°C in a 5% CO_2_ atmosphere. After incubation for 1h, cells were washed with PBS and processed for further analysis. For immunofluorescence staining cells were grown in ibidi 96-well μ-plates (Ibidi GmbH, Munich, Germany) and for Western blotting in 24-well plates (Cellstar ^®^, Greiner Bio-One GmbH, Austria).

To assess the binding of different concentrations of ORF2-3 to LMH cells, cells were harvested and subjected to Western blotting analysis as described above. Additionally, blocking of ORF2-3 binding to LMH cells was performed following the same binding condition as applied above, except that ORF2-3 was pre-incubated with 1:50 diluted chicken serum raised against recombinant ORF2-3 or a negative serum as control for 1h at RT. The chicken sera were obtained either from a non-inoculated specific pathogen free (SPF) bird or from an SPF bird inoculated with recombinant ORF2-3 together with GERBU Adjuvant LQ no. 3000 (GERBU Biotechnik GmbH, Heidelberg, Germany). Chicken anti-sera were raised in our own laboratory (license number: BMWFW-68.205/0158-WF/V/3b/2014).

Amersham^™^ ECL^™^ Prime Western Blotting Detection Reagent (GE Healthcare, Little Chalfont, Bukinghamshire, UK) was applied for semi-quantitative analysis and SuperSignal^™^ West Pico Chemiluminescent Substrate (Pierce, Thermo Fisher Scientific, Life Technology, Carlsbad, CA, USA) was used for qualitative detection

### Immunofluorescence staining

Following the binding assay described above, cells were fixed with methanol for 5min at -20°C. After washing with PBS, cells were blocked with 3% BSA in PBS for 1h at RT and incubated with mouse monoclonal anti-Xpress (1:2000, Invitrogen, Life Technologies, Carlsbad, CA, USA) overnight at 4°C. After washing, cells were incubated with Alexa Fluor 488-conjugated goat anti-mouse IgG (1:2000, Invitrogen, Life Technologies, Carlsbad, CA, USA) for 1h at RT. After washing off the secondary antibody, cells were stained with 4'6-diamidino-2-phenylindole (DAPI; Roche Diagnostics GmbH, Vienna, Austria) and Whole Cell Stain (Invitrogen, Life Technologies, Carlsbad, CA, USA). Following a final washing step, cells were mounted in ibidi Mounting Medium (Ibidi GmbH, Munich, Germany). Binding and internalization of proteins were examined using a Zeiss Axiovert 200 M fluorescence microscope or a Zeiss 510 Meta confocal microscope (Zeiss, Jena, Germany).

### Influence of heparin sodium salt on the binding

ORF2-3 at 500nM was pre-incubated with heparin sodium salt (Sigma-Aldrich, Steinheim, Germany) at concentrations of 0.001μg/ml to 10μg/ml at RT for 30 min. Untreated ORF2-3 and ORF2-4 were used as controls. Both heparin sodium salt pretreated and untreated proteins were used in binding assay with LMH cells as described above.

All samples were tested in duplicates by immunofluorescence staining. For each well, three images were taken and the mean fluorescence intensity of cells was measured with ImageJ software (http://imagej.nih.gov/ij/download.html). Fluorescence intensity values of ORF2-3 bound to LMH cells in the absence of inhibitors was considered as 100% and the others were expressed relative to it. Additionally, samples were examined by Western blotting as described above.

### Influence of cellular sulfation on the binding

To reduce cellular sulfation, LMH cells were cultured for 48h to 96h in medium containing 20mM to 40mM sodium chlorate (Sigma-Aldrich, Steinheim, Germany). Control samples were cultivated without the addition of sodium chlorate. All cells were gently washed and prossessed in the binding assay as described above, analyzed by immunofluorescence staining and ImageJ as described for the experiments with heparin sodium salt.

### Heparinase II treatment of LMH cells

LMH cells were pre-treated in Heparinase Reaction Buffer (20 mM Tris-HCl, 100 mM NaCl and 1.5 mM CaCl_2_) with heparinase II (10U/ml) from *Bacteroides* (New England Biolabs GmbH, Frankfurt am Main, Germany) or PBS as control for 2h at 37°C in an atmosphere supplied with 5% CO_2_. After 2h of incubation cells were washed with PBS and incubated with ORF2-3 or ORF2-4 (500nM each) for 1h at 37°C with 5% CO_2_. Samples were analyzed by immunofluorescence staining as described for the experiments with heparin sodium salt.

## Results

### Expression and preparation of truncated avian HEV capsid proteins

**Fig 1:**
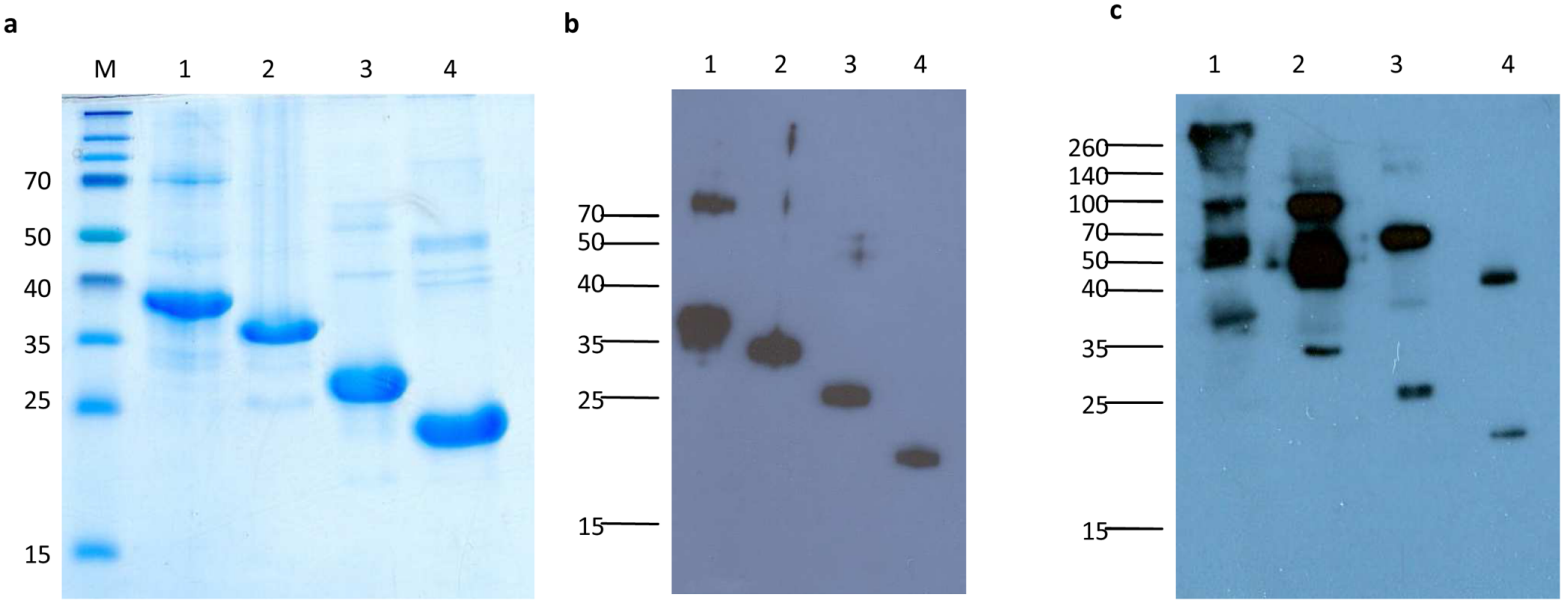
Expression and preparation of truncated avian HEV capsid proteins. (a) Purified and refolded recombinant proteins were separated by reducing SDS-PAGE and visualized with Coomassie blue. M, marker (Thermo Scientific, Vienna, Austria); lane 1 to lane 4, ORF2-1, ORF2-2, ORF2-3 and ORF2-4. Western blotting analysis of refolded recombinant proteins with anti-His antibodies were performed following SDS-PAGE separation under (b) reducing or (c) non-reducing conditions, lane 1 to 4, ORF2-1, ORF2-2, ORF2-3 and ORF2-4.

The N-terminal 6xHistidine- and Xpress- tagged recombinant proteins of avian HEV capsid proteins ORF2-1, ORF2-2, ORF2-3 and ORF2-4 (S1 Fig) were successfully expressed as inclusion bodies. Refolded and dialyzed truncated proteins were detected with the size of approximate 36KDa, 34KDa, 26KDa and 20KDa, respectively, by reducing SDS-PAGE (Fig 1a) and based on the reducing SDS-PAGE, truncated proteins were confirmed by Western blotting (Fig 1b).

In order to investigate whether refolded proteins are able to establish homo-oligomeric forms, truncated proteins were separated by SDS-PAGE under non-reducing condition and transferred to PVDF membrane for Western blotting analysis. Oligomeric forms of truncated proteins were observed. Highest molecular weight of ORF2-1oligomers observed in Western blotting analysis was more than 260KDa and trimers of ORF2-3 and ORF2-4 were revealed with molecular weight of about 70KDa to 50KDa, respectively (Fig 1c).

### Binding of truncated proteins to LMH cells

**Fig 2:**
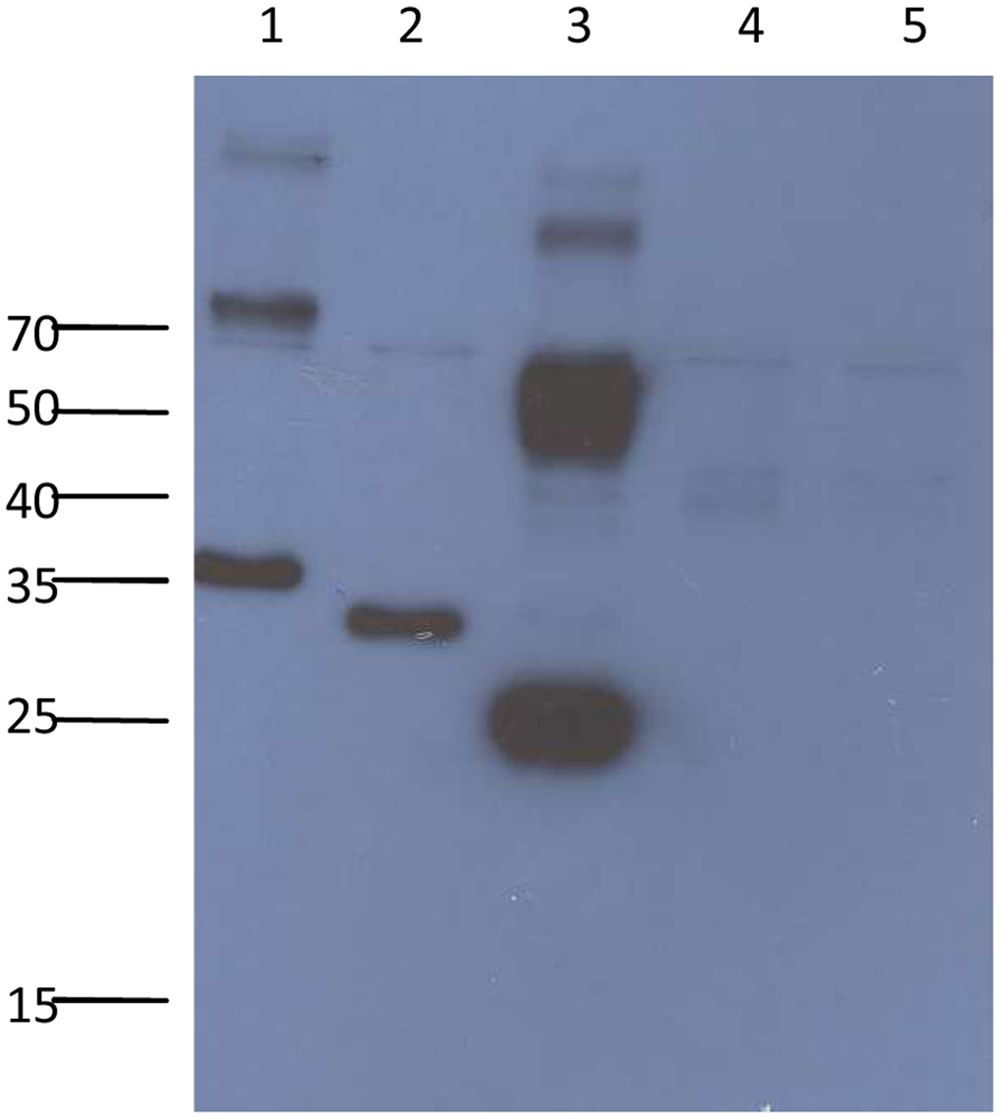
Western blotting analysis of binding assay with LMH cells. Lanes 1 to 4, ORF2-1, ORF2-2, ORF2-3 and ORF2-4, respectively. Lane 5, PBS as negative control.

To map the region essential for binding to host cells, the binding assay utilizing truncated avian HEV capsid proteins ORF2-1, ORF2-2, ORF2-3 and ORF2-4 to LMH cells was performed. Western blotting analysis resulted in positive bands for ORF2-1, ORF2-2 and ORF2-3, whereas no signal could be detected for the ORF2-4 construct and the negative control (Fig 2).

**Fig 3:**
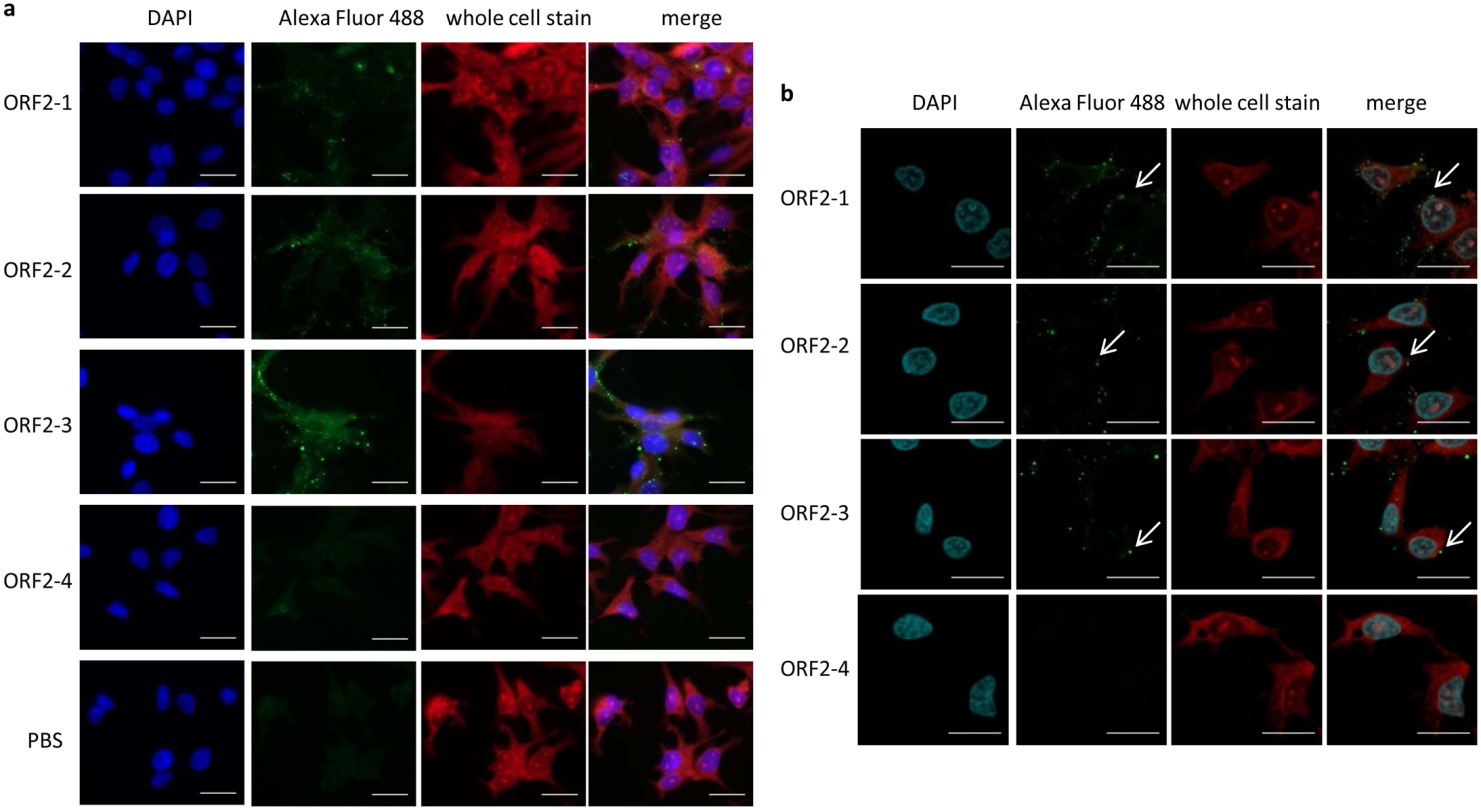
Indirect immunofluorescence staining to investigate binding to LMH cells. Cells were incubated with 500nM recombinant proteins, each, for 1h and fixed, then stained with anti-Xpress antibodies, followed by Alexa Fluor 488-conjugated anti-mouse IgG. Cells were co-stained with DAPI (blue) and Whole Cell Stain (red), afterwards imaged using (a) widefield fluorescence or (b) confocal microscopy. PBS was used as negative control. Arrows indicate the location of proteins binding to the surface of LMH cells. Bar, 20μm.

The binding of ORF2-1, ORF2-2 and ORF2-3 to LMH cells was further confirmed by immuofluorescence staining (Fig 3a). No signal was detected with the ORF2-4 construct and the negative control. Cells incubated with ORF2-1, ORF2-2 and ORF2-3 were examined by confocal microscopy to investigate the internalization of the truncated proteins. Images showed that all proteins capable of binding to LMH cells were localized on the cell surface, and no internalization could be detected (Fig 3b, examples indicated by arrows).

**Fig 4:**
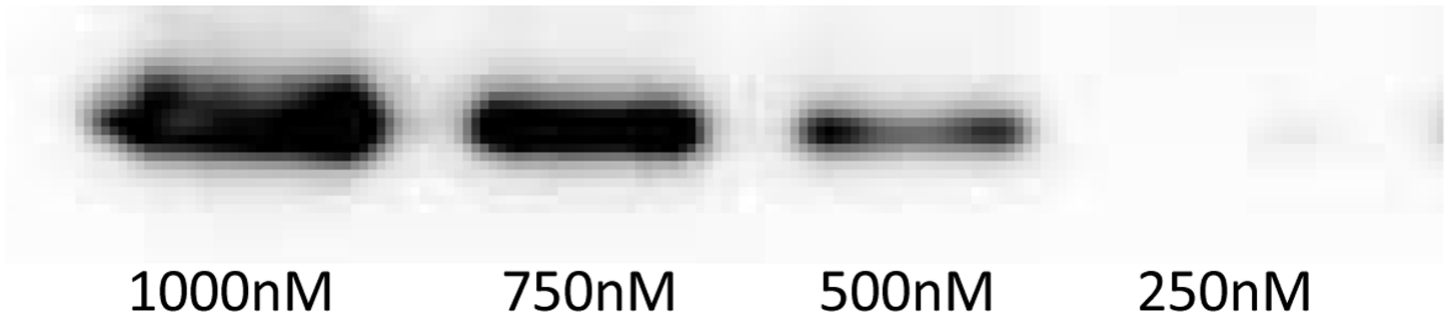
Semi-quantification of ORF2-3 binding to LMH cells. Different concentrations of ORF2-3 were incubated with cells for 1h. Binding was analyzed by Western blotting applying Amersham™ ECL™ Prime Western Blotting Detection Reagent.

To determine whether binding of the capsid protein to LMH cells is concentration dependent, the ORF2-3 construct was applied to LMH cells in concentrations ranging from 250nM to 1000nM. Western blotting analysis demonstrated that binding declined with a decreasing amount of protein. At a concentration of 250nM, binding of ORF2-3 to LMH cells was undetectable (Fig 4).

### Binding of ORF2-3 to QT-35 and HepG2 cells

**Fig 5:**
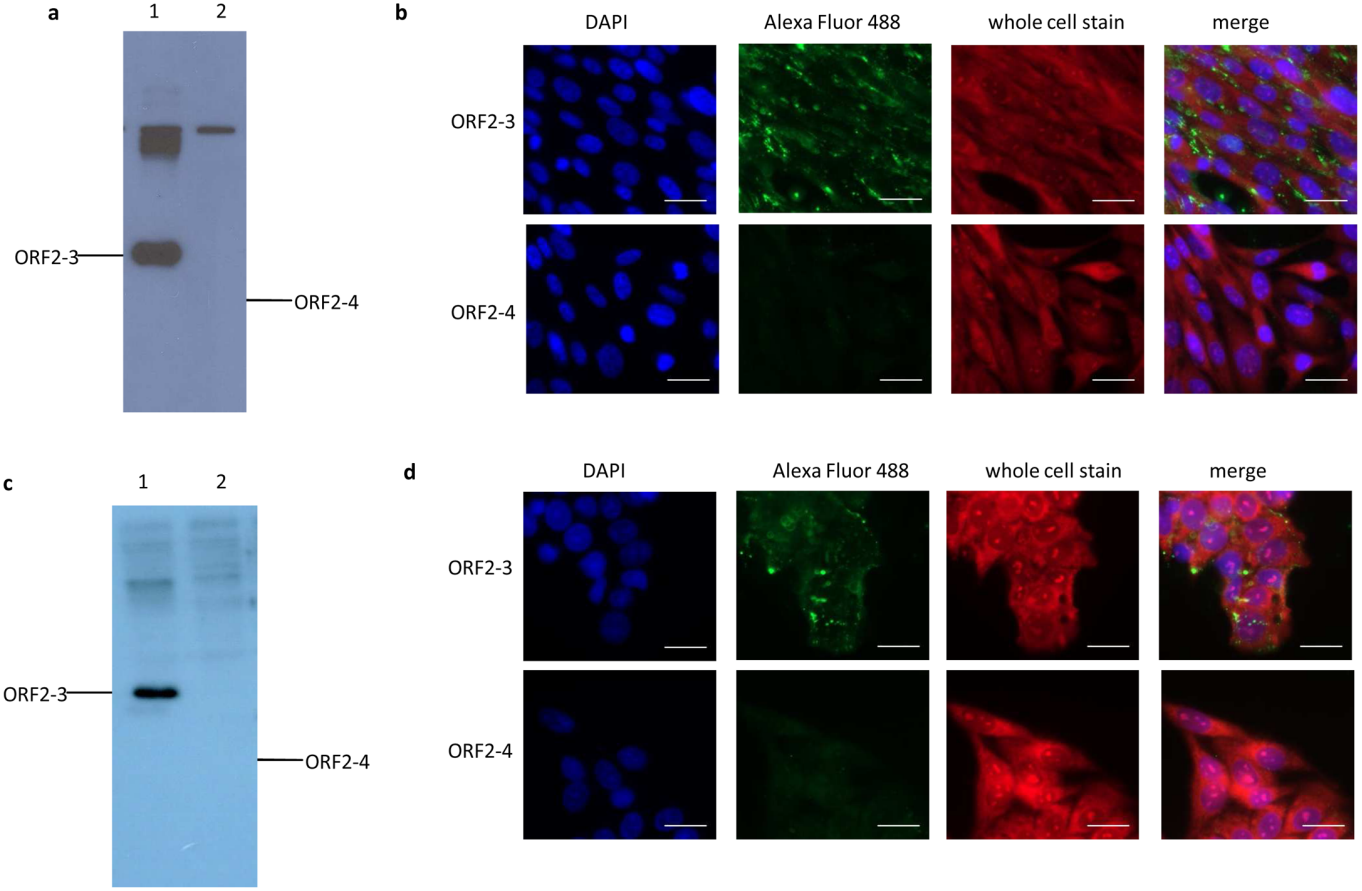
Binding of ORF2-3 to QT-35 and HepG2 cells. Western blotting analysis of ORF2-3 binding to (a) QT-35 and (c) HepG2 cells. Immunofluorescence staining of ORF2-3 binding to (b) QT-35 and (d) HepG2 cells. ORF2-4 was used as a negative control. Bar, 20μm.

To explore the potential of avian HEV to attach to cells from other species, ORF2-3 and ORF2-4 constructs were used in binding assays with QT-35 and HepG2 cells. Western blotting analysis showed that ORF2-3 bound to both cell lines, whereas no binding of ORF2-4 to any cell line was noticed (Fig 5a and 5c). An unspecific band with higher molecular weight than ORF2-4 was observed in the binding assay of ORF2-4 to QT-35 cells which appeared in binding of ORF2-3 to QT-35 cells as well and partially overlapped with the signal of ORF2-3 oligomer (Fig 5a). Binding of ORF2-3 to both cells was further confirmed by immunofluorescence staining. The fluorescent signal could be detected in assays using the ORF2-3, whereas the application of ORF2-4 resulted in no signal (Fig 5b and 5d).

### Binding specificity of ORF2-3

**Fig 6:**
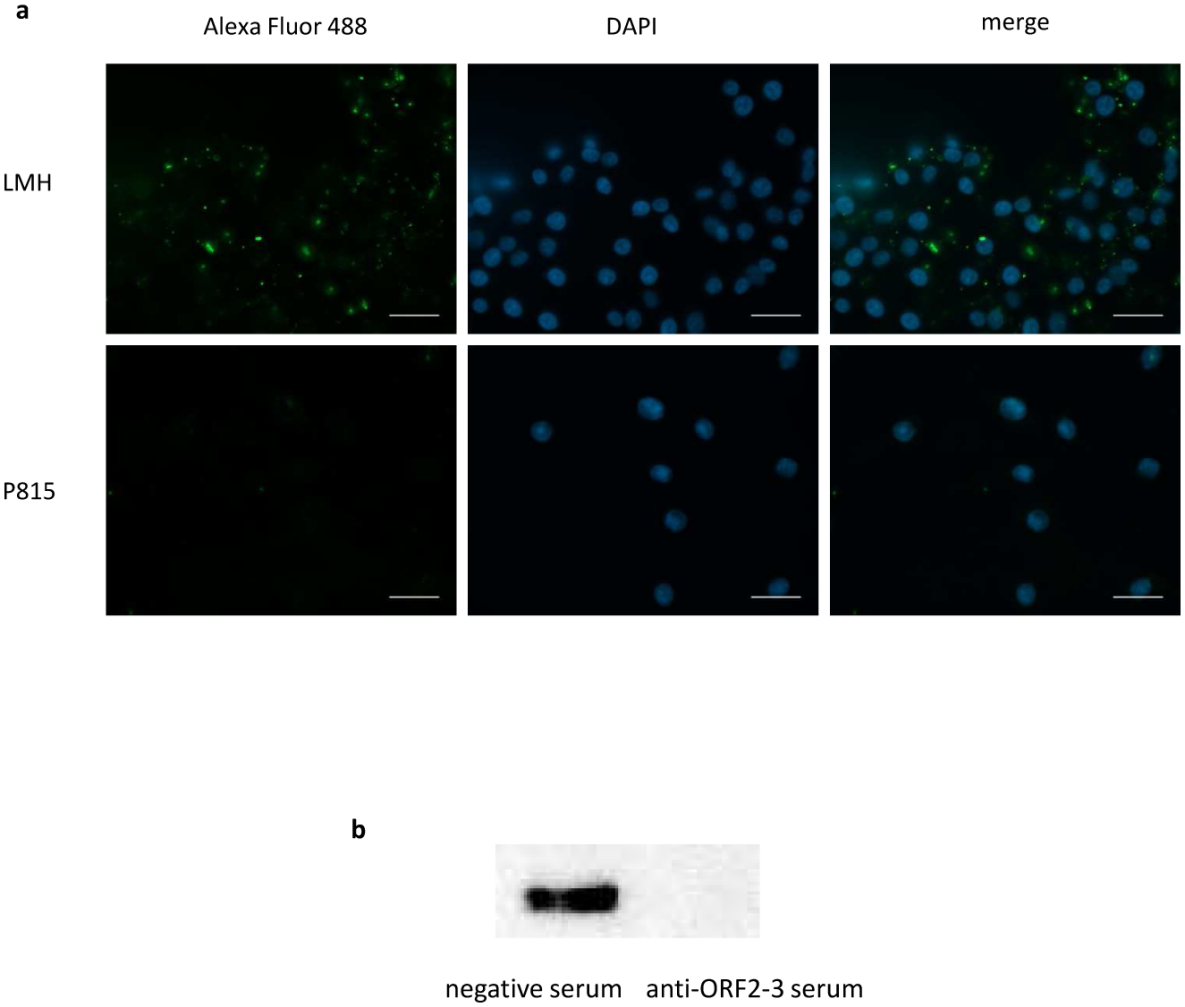
Investigating binding specificity. (a) Immunofluorescence staining of ORF2-3 binding assay to LMH and P815 cells. Bar 20μm. (b) Western blotting analysis applying negative control serum in comparison to chicken serum raised against ORF2-3 to block the interaction with LMH cells.

To assess the binding specificity of ORF2-3, P815 cells were used in the binding assay. Fluorescence staining demonstrated that ORF2-3 could not attach to mouse mastocytoma P815 cells in contrast to the positive staining signals retrieved in the binding assay applying ORF2-3 to LMH cells (Fig 6a).

In order to further confirm the specificity of binding, blocking of the binding process was carried out with a chicken serum raised against ORF2-3. The results showed successful blocking of ORF2-3 binding to LMH cells by pre-treatment of ORF2-3 with a chicken anti-serum in comparison to a negative control serum (Fig 6b).

### Effect of heparin sodium salt on binding of ORF2-3 to LMH cells

**Fig 7:**
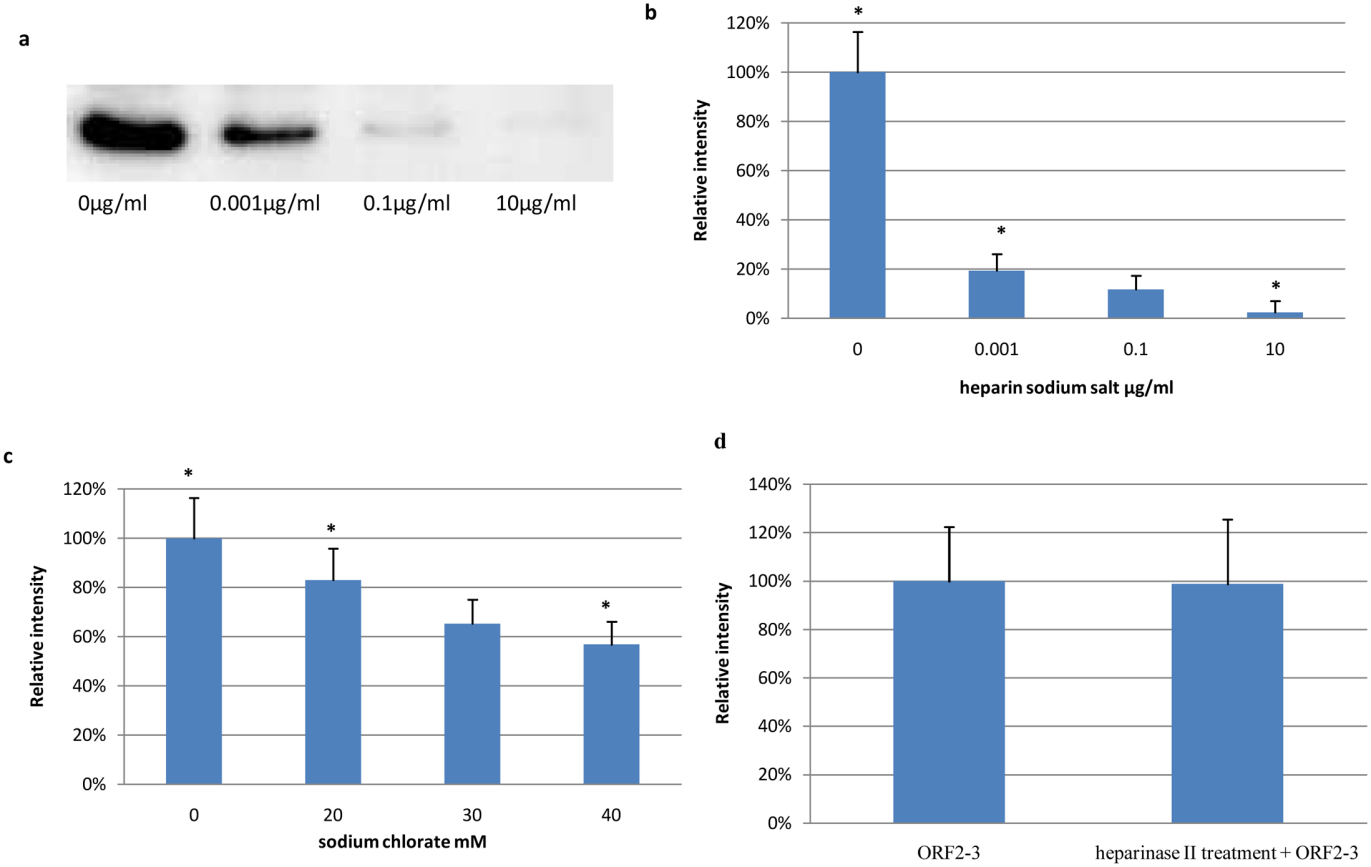
Effect of heparin sodium salt, sodium chlorate and heparinase II treatment in binding assay. (a) Western blotting analysis following treatment with heparin sodium salt. Relative intensity of immunofluorescence signal derived from different binding assays (b) heparin sodium salt, (c) sodium chlorate and (d) heparinase II. Mean fluorescence intensity of stained cells was measured with ImageJ. Error bars represent standard deviation. *, p < 0.05.

To determine whether heparin plays a role in the interaction between avian HEV and host cells, ORF2-3 was incubated with different concentrations of heparin sodium salt prior to the incubation with cells. Binding assay analyzed by Western blotting demonstrated that heparin sodium salt significantly inhibited the binding of ORF2-3 to LMH cells in a dose-dependent manner (Fig 7a). This result was further confirmed by immunofluorescence staining and a similar effect was observed (Fig 7b).

### Effect of sodium chlorate on binding of ORF2-3 to LMH cells

To investigate the importance of host cell proteins sulfation on binding of the capsid protein, LMH cells were cultured in medium containing 0mM to 40mM sodium chlorate to inhibit the sulfation reaction. A dose-dependent decrease in binding of ORF2-3 upon the treatment of cells with sodium chlorate was observed (Fig 7c). The addition of sodium chlorate (20mM to 40mM) in the cell culture medium prior to binding assay resulted in the inhibition of the ORF2-3 binding with a mean percentage ranging from 17.0% to 43.2%, respectively.

### Effect of heparinase II treatment of LMH cells on binding of ORF2-3

In order to test whether avian HEV attaches to host cells via heparan sulfate proteoglycans (HSPGs), cells were treated with heparinase II. For that purpose LMH cells were incubated with or without heparinase II (10U/ml) for 2h at 37°C before the addition of ORF2-3 or ORF2-4. In contrast to the effect of heparin sodium salt and sodium chlorate treatments, heparinase II treatment of LMH cells had no significant effect on binding of ORF2-3 (Fig 7d). Again, no binding of ORF2-4 could be recorded. Efficiency of heparinase II was demonstrated by adding into the mixture of the ORF2-3 and the heparin sodium salt prior to the binding assay which reverted the inhibitory effect of heparin sodium salt on the binding of ORF2-3 to LMH cells (data not shown).

## Discussion

Binding of a certain virus to host cells represents a crucial step in the infection process. The present study describes the establishment of a system to investigate virus-host interaction of avian HEV, employing recombinant capsid protein construct(s). Due to the lack of an efficient *in vitro* cell culture system to study avian HEV, host-pathogen interaction studies using the infectious virus are fairly limited. The use of recombinant truncated capsid proteins of mammalian HEV to explore the interaction between virus and host cells was investigated in several studies. These studies demonstrated that the C-terminal region of the capsid protein, as protruded spike on the surface of virus particles, resembles putative binding sites for both cellular receptors and neutralizing antibodies [16,30]. Therefore, in the present study, the binding ability of truncated recombinant avian HEV capsid protein ORF2-1 (aa339-606) and its 3 shorter constructs with gradual C-terminal deletion was investigated. The shortest construct, ORF2-4, consisting of amino acids 339–470, did not bind to any of the investigated cell lines. This construct lacks 37 C-terminal amino acids as compared to ORF2-3, the nearest construct that demonstrated an efficient binding. This result suggests that the region from amino acids 471 to 507 of the avian HEV capsid protein plays a crucial role in the attachment to cells. The importance of the C-terminus of the capsid protein for virus-host interaction is in agreement with findings reported for mammalian HEV capsid protein [23,24,26]. It needs to be kept in mind that avian and mammalian HEV capsid proteins vary not only by size but also in amino acids sequence similarity [3]. Even though avian HEV and mammalian HEV share common features, amino acid identity between these proteins is only 50% and avian HEV capsid protein has its own unique epitope [3,31].

Previous studies on human HEV indicated that glycan moieties might not be crucial for the viral attachment based on experiments with recombinant proteins derived from *E*. *coli* expression system [24]. In agreement with human HEV sequence analyses of avian HEV suggested potential N-linked glycosylation sites, ^255^NLS and ^522^NGS [3,12]. Despite the fact that an *E*. *coli* expression system was applied in the actual investigations all constructs omitted the ^255^NLS glycosylation site whereas ^522^NGS was not included in the shortest construct (ORF2-3) for which binding could still be demonstrated.

Despite their binding to cells, the internalization of truncated capsid proteins was not observed in this study and extension of the N-terminal part of the capsid protein might be needed to induce internalization. This has been reported for recombinant human HEV capsid protein p239 corresponding to avian HEV capsid protein aa313-552 [23]. However, it cannot be excluded that avian HEV uses a different pathway for cell entry; since in contrast to mammalian HEV, heparinase II had no influence on binding to host cells (see below).

In the present study, the same binding pattern of ORF2 constructs could be observed in assays with QT-35 and HepG2 cells of Japanese quail and human origin, respectively. This indicates that avian HEV might be able to attach to cells of another bird species, and even humans. The attachment to cells from other bird species and even a possible infection of other birds are supported by recent epidemiological studies based upon the presence of antibodies [32–34]. Furthermore, it has been demonstrated that avian HEV originating from chickens is capable to infect turkeys [35]. In contrast to these findings in birds, the zoonotic potential of avian HEV remains very low. This has its roots in the unsuccessful experimental attempt to infect rhesus monkeys with avian HEV [13] and the fact that an infection in humans was never reported.

It has been reported that HSPGs, specifically syndecans, play an important role in the binding of mammalian HEV capsid protein to cells [25]. Furthermore, *in vitro* infection experiments confirmed the involvement of HSPGs, as the enzymatic treatment of cells demonstrated a significant reduction in human HEV infection [25]. In the actual study, pre-incubation of ORF2-3 with heparin sodium salt efficiently inhibited the binding of ORF2-3 to LMH cells. In addition, the treatment of cells with sodium chlorate induced inhibition of ORF2-3 binding to about 50%. These results are in agreement with studies of human HEV construct [25], and might implicate the involvement of HSPGs in the attachment of avian HEV capisd protein as well. However, since the heparinase II treatment of LMH cells did not significantly influence the binding of the ORF2-3, the HSPGs can be neglected as main targets involved in the attachment of avian HEV capsid protein. The fact that heparin sodium salt and sodium chlorate treatments significantly impaired the binding of the ORF2-3 to LMH cells suggests that sulfated molecule(s) with heparin-like structure are required for the attachment of avian HEV capids protein to the cell surface. Possible candidates might be dermatan sulfate proteoglycans, which are shown to efficiently bind many proteins that can interact with heparin [36]. In this context it needs to be considered that earlier studies suggested for human HEV the involvement of different receptor-binding sites within the capsid protein [23], with a certain importance of the C-terminal region [25]. Anyhow, the possibility that the N-terminus alone or together with the C-terminus is involved in receptor-binding sites could not be excluded.

In conclusion, we have successfully mapped the capsid protein region from amino acid 471 to 507 as critical for the attachment of avian HEV to host cells. Three truncated constructs of the avian HEV capsid protein; ORF2-1, ORF2-2 and ORF2-3, bound to LMH cells and the shortest one, ORF2-3, was selected for further binding studies between virus and other potential host cells. It could be demonstrated that ORF2-3 was capable to bind to QT-35 and HepG2 cells, indicating a potential of avian HEV to attach to cells of species other than chicken. Treatments with heparin sodium salt or sodium chlorate significantly reduced the binding of ORF2-3 to cells. However, treatment with heparinase II had no obvious effect on the ORF2-3 binding. This suggests that avian HEV might utilize a different cellular receptor(s) for the attachment to cells as compared to mammalian HEV, an issue that needs to be addressed in future studies.

## Supporting Information

S1 Fig Schematic diagram of truncated recombinant avian HEV capsid protein constructs. (PPTX)

S1 Table List of primers to construct truncated recombinant capsid proteins. (DOCX)
