# Supplementary figures and images for: C-Terminal Amino Acids 471-507 of Avian Hepatitis E Virus Capsid Protein Are Crucial for Binding to Avian and Human Cells
Source: PLoS One. 2016 Apr 13;11(4):e0153723. doi: 10.1371/journal.pone.0153723 (PMC4830555; doi:10.1371/journal.pone.0153723)

## Slide 1
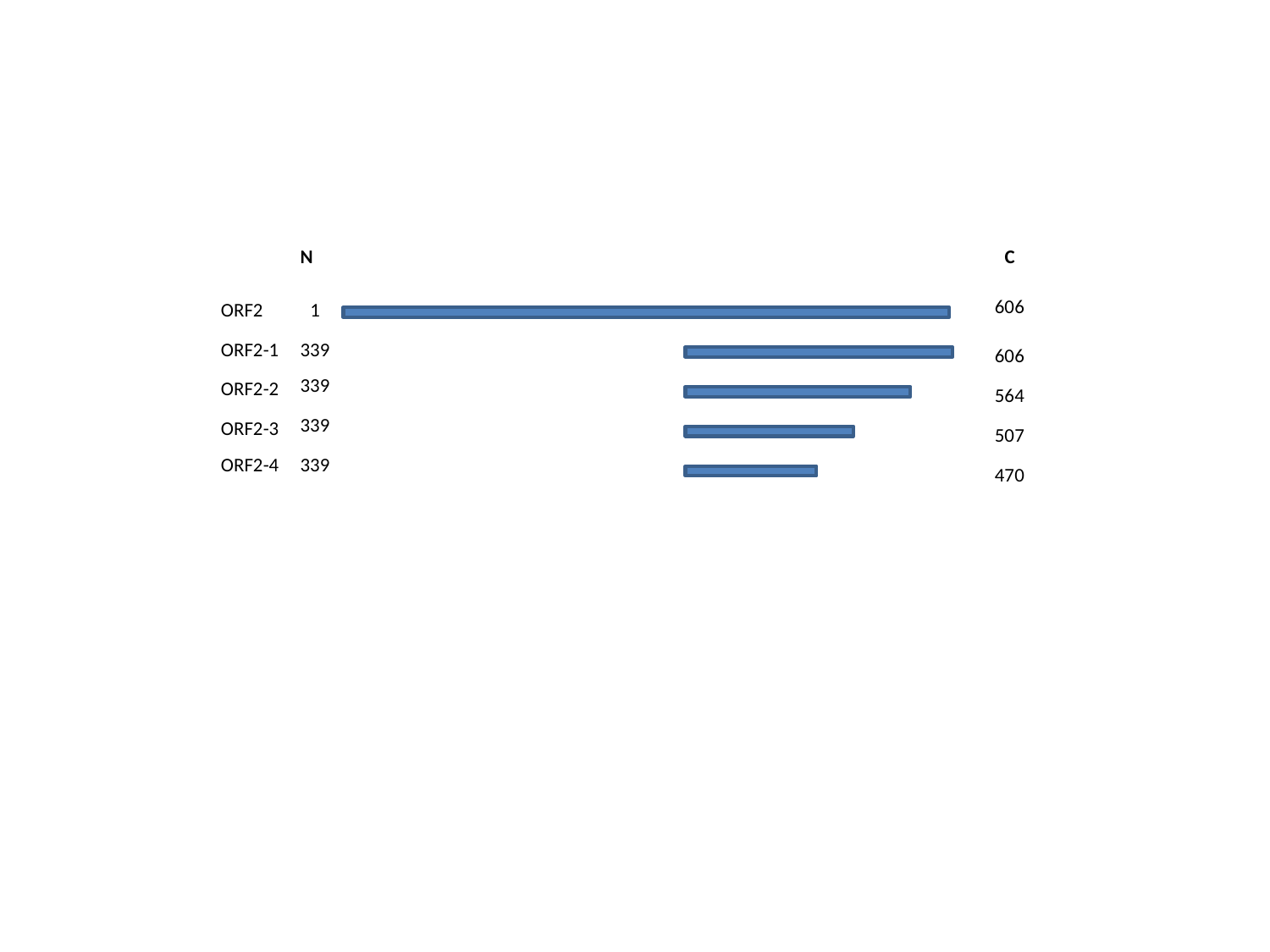

N
C
606
ORF2
1
ORF2-1
339
606
339
ORF2-2
564
339
ORF2-3
507
ORF2-4
339
470

Supplement: S1 Fig — (PPTX) [file pone.0153723.s001.pptx]
