# Supplementary material for: C-Terminal Amino Acids 471-507 of Avian Hepatitis E Virus Capsid Protein Are Crucial for Binding to Avian and Human Cells
Source: PLoS One. 2016 Apr 13;11(4):e0153723. doi: 10.1371/journal.pone.0153723 (PMC4830555; doi:10.1371/journal.pone.0153723)
Supplement: S1 Table — (DOCX) [file pone.0153723.s002.docx]

**S1 Table. List of primers to construct truncated recombinant capsid proteins**

| Gene | Primers |
| --- | --- |
| ORF2-1 | Forward: 5'-AGATCTCAGTATATGTACGGCCGGCCTG-3' |
|  | Reverse: 5'-AAGCTTTTAGGGTGGTGAGGGGAATGT-3' |
| ORF2-2 | Reverse: 5'-AAGCTTTTAACCGGTGTGATGCGCCAGAAA-3' |
| ORF2-3 | Reverse: 5'- AAGCTTTTAATAAAAGTACCCGGCGCCCTG-3' |
| ORF2-4 | Reverse: 5'- AAGCTTTTAATCCACAGTAGCCTTAGTCCA-3' |

Restriction sites and stop codons are underlined
